# Targeting Inflammation by Anthocyanins as the Novel Therapeutic Potential for Chronic Diseases: An Update

**DOI:** 10.3390/molecules26144380

**Published:** 2021-07-20

**Authors:** Aleksandra Kozłowska, Tomasz Dzierżanowski

**Affiliations:** Department of Social Medicine and Public Health, Medical University of Warsaw, 02-776 Warsaw, Poland; akozlowska2@wum.edu.pl

**Keywords:** anthocyanins, inflammation, oxidative stress

## Abstract

Low-grade chronic inflammation (LGCI) and oxidative stress act as cooperative and synergistic partners in the pathogenesis of a wide variety of diseases. Polyphenols, including anthocyanins, are involved in regulating the inflammatory state and activating the endogenous antioxidant defenses. Anthocyanins’ effects on inflammatory markers are promising and may have the potential to exert an anti-inflammatory effect in vitro and in vivo. Therefore, translating these research findings into clinical practice would effectively contribute to the prevention and treatment of chronic disease. The present narrative review summarizes the results of clinical studies from the last 5 years in the context of the anti-inflammatory and anti-oxidative role of anthocyanins in both health and disease. There is evidence to indicate that anthocyanins supplementation in the regulation of pro-inflammatory markers among the healthy and chronic disease population. Although the inconsistencies between the result of randomized control trials (RCTs) and meta-analyses were also observed. Regarding anthocyanins’ effects on inflammatory markers, there is a need for long-term clinical trials allowing for the quantifiable progression of inflammation. The present review can help clinicians and other health care professionals understand the importance of anthocyanins use in patients with chronic diseases.

## 1. Introduction

Inflammation affects a wide variety of physiological and pathological processes. This condition is an essential component of immune surveillance and host defense. Although the pathological aspects of numerous mechanisms of inflammation are well recognized, their physiological functions are mostly unexplained.

Low-grade chronic inflammation (LGCI) is a pathological feature of a wide range of chronic conditions. LGCI is characterized by elevated concentrations of inflammatory markers in the absence of any overt symptoms. However, this condition has not yet been consistently defined or measured. Although there is likely a genetic predisposition, many other triggers can impact the inflammatory process. Some exogenous and endogenous factors such as smoking, air pollution, silica dust, recurrent episodes of acute inflammation, persistent infections, autoimmune disorders, and overweight or obesity have been identified. Several triggers including overproduction of reactive oxygen species (ROS) and advanced glycation end products (AGEs), mitochondrial dysfunction, renin–angiotensin system (RAS) deregulation, hormonal changes, uric acid (urate) crystals, oxidized lipoproteins, homocysteine, visceral adiposity, an imbalance in the gut microbiota, and accumulation of cell debris due to defective autophagy also play a significant role [1,2]. 

In recent years LGCI has been shown to contribute to most if not all chronic diseases typical of age-related decline of many functional systems in the older population. This phenomenon has been termed “inflamm-aging” [1,3]. LGI has also been given the name “metaflammation” (an inflammation of metabolic tissue), which originates from metabolic cells in response to excess nutrients [4]. There is a general lack of sensitive and specific biomarkers of low-grade chronic inflammation that can be used in human trials. In humans, the most well-accepted markers of systemic inflammation are a number of circulating pro-inflammatory cytokines like tumor necrosis factor-alpha (TNF-α), interleukin-1 beta (IL-1β), interleukin-6 (IL-6), and interleukin-8 (IL-8). To date, high-sensitivity C-reactive protein (hsCRP), fibrinogen, and such cellular biomarkers like the white blood cell and platelet counts have also been used to assess LGCI [5,6,7].

Current research provides links among the change in inflammatory profile and the risk of a number of chronic conditions, including metabolic syndrome (MetS), non-alcoholic fatty liver disease (NAFLD), type 2 diabetes mellitus (T2DM), cancer, cardiovascular, and neurodegenerative disease [4,8,9,10,11,12].

Inflammation and oxidative stress act as cooperative and synergistic partners in the pathogenesis of a wide variety of diseases, elevating adverse chronic diseases’ risk factors levels [13]. As an example, misdirected oxidative stress in various tissues potentiates inflammatory responses and inciting target organ damage [14]. Oxidative stress occurs when an organism accumulates more ROS than can be eliminated by antioxidant defense mechanisms. The accumulation of ROS and free radicals in a cell affects many important compounds, such as lipids, proteins, DNA, carbohydrates, and enzymes, and can result in cell damage [15]. In healthy humans, cells defend themselves against ROS-related damage through antioxidants that prevent or counterbalance oxidation even at low concentrations. The ROS and antioxidant protection against free radical tissue injury are in balance [16]. It has been reported that impaired oxidant—antioxidant status is involved in the etiopathogenesis of various complications [17,18,19]. 

Dietary factors are involved in regulating the inflammatory state and activating the endogenous antioxidant defenses. Protective dietary compounds such as polyphenols, which are consumed together with a human diet, may be beneficial in attenuating the potentially harmful risk factors of chronic diseases. Among them, anthocyanins are emerging potential agents for counteracting the onset and progression of numerous non-communicable diseases such as neurodegenerative, cardiovascular, and metabolic diseases and cancer. They have been shown to stimulate immunomodulatory and antioxidant effects, thereby blunting the cooperative and synergistic deleterious effects of oxidative stress and pro-inflammatory cytokines and may, therefore, provide protection against chronic diseases [20,21]. Their anti-inflammatory activity has been widely investigated by many authors. In the query performed in the ClinicalTrials.gov database in May 2021, the keyword “anthocyanin” has identified 145 clinical studies in which anthocyanins were registered.

## 2. Methodology

We searched Medline via PubMed database for clinical studies to evaluate the effects of anthocyanins on pro-inflammatory cytokines and oxidative stress parameters. We used the following query:

(anthocyanins[Title/Abstract] OR pelargonidin[Title/Abstract] OR cyanidin[Title/Abstract] OR peonidin[Title/Abstract] OR delphinidin[Title/Abstract] OR petunidin[Title/Abstract] OR malvidin[Title/Abstract] OR cyanidin-3-*O*-glucoside[Title/Abstract]) AND (inflammation[Title/Abstract] OR inflammatory[Title/Abstract] OR inflammatory mediators[Title/Abstract] OR oxidative stress[Title/Abstract])

Using the filters, we limited the article types to clinical trials, meta-analysis, or randomized controlled trials and the publication date range from 1 January 2016 to 10 June 2021. As a result, we retrieved 29 clinical papers for full-text reading.

We did not apply any publication time restrictions for preclinical data. However, we presented an update of the most recent articles (published in 2020) with which the readers might be unfamiliar.

## 3. Anthocyanins and Their Metabolites

Chronic inflammation may be influenced by diet, with an inestimable role for dietary habits. There is evidence to support specific dietary strategies to modulate LGCI. Both supplementations and habitual intake of certain food compounds such as anthocyanins seem to be beneficial. Anthocyanins constitute a subclass of flavonoids with more than 700 structurally different anthocyanin derivatives of 27 aglycons identified. Anthocyanins are glucosides of the anthocyanidins (precursors of anthocyanins), which are derivatives via the phenylpropanoid pathway. Due to their multiple phenyl groups, anthocyanins are rarely found as aglycons (anthocyanidins). The aglycone part of the anthocyanins is unstable and quite reactive due to the electron-deficient flavylium cation and has lower solubility, so it occurs almost exclusively in the glycosylated form (as anthocyanins). Glycosylation helps maintain the hydrophilicity and stability of hydrophobic flavonoids [22,23].

**Figure 1:**
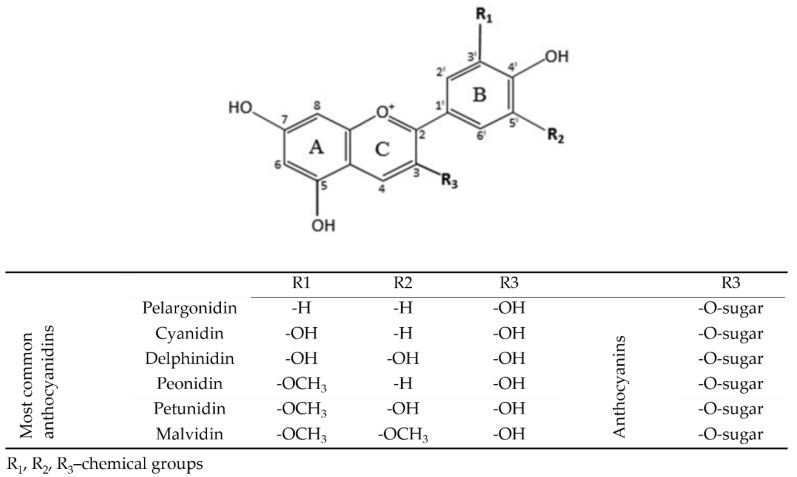
Chemical structure of the main anthocyanidins and anthocyanins present in plant food; based on [16,24,25].

Common anthocyanins are composed of one of six anthocyanidin bases, which differ in molecular structure at the B-ring and a sugar moiety attached at the third position of the C-ring (Figure 1). The six predominant molecules in plants, representing approximately 90% of all anthocyanidins known to date, are pelargonidin, cyanidin, peonidin, delphinidin, petunidin, and malvidin.

## 4. Food Sources and Dietary Intake

Anthocyanins are responsible for the production of red-orange to blue-violet pigments in plants. Important dietary sources of these substances are fruits such as berries, currants, grapes, and some tropical fruits. Leafy vegetables, grains, roots, and tubers show very high anthocyanins concentration as well (Table 1). It is important to note that particular anthocyanin concentrations in plants depend on genetic, environmental, and agronomic factors such as light, temperature, humidity, fertilization, stage of maturity at harvest, food processing, and storage conditions, as well as the specific plant morphological component. Differences in flavonoid content between plant species are usually small [22].

There are several databases of anthocyanin contents in foods, which can be used to evaluate daily intake. The most commonly used are the U.S. Department of Agriculture (USDA) databases (https://fdc.nal.usda.gov/ accessed on 19 July 2021) and the online Phenol-Explorer database (https://phenol-explorer.eu accessed on 19 July 2021). Both of them contain improved food flavonoid composition estimates for both raw and cooked items by type of cooking method. It should be noted that USDA databases, in general, are compiled from analysis utilizing chromatography after hydrolysis (which removes the glycosides present in the food matrix and enables total quantification of the aglycone equivalents) while Phenol-Explorer collects data that were mostly measured utilizing chromatography without hydrolysis, in which each individual flavonoid form, as found in food, is quantified [26]. However, these databases are still unlikely to be representative of all foods consumed, mainly due to the incomplete data on the anthocyanin quantities in food. The recently increased availability of food composition data for flavonoids has allowed for measuring the amount of intake with greater accuracy [27]. However, a comparison of the results of nutritional assessments of human diet obtained on the basis of the various data sources may differ and be inaccurate [28].

Anthocyanins are not essential nutrients, and the majority of global dietary guidelines have not defined a daily intake for these substances. However, Dietary References Intake published by the Chinese Nutrition Society suggested a specific proposed level (SPL) as 50 mg per day of these substances [30,31]. Moreover, both recommendations to increase the consumption of colorful fruits and vegetables and contemporary dietary patterns such as Mediterranean-Dash Intervention for Neurodegenerative Delay (MINDs) Diet help promote the consumption of bioactive compounds such as anthocyanins [32,33]. These substances are authorized by the European Food Safety Agency (EFSA) as food additives in the European Union as E163. However, acceptable daily intake (ADI) has not been established by the EFSA scientific panel due to insufficient toxicological research [34]. Nevertheless, the Joint FAO/WHO Expert Committee on Food Additives (JECFA) has established an ADI of 2.5 mg/kg of body weight per day for anthocyanins [22]. Furthermore, it is important to mention that, taking into account the estimated exposure to anthocyanins in the European population, anthocyanins as food additives seem to be safe [34].

The daily dietary intake of anthocyanins has been estimated at about 11.6 ± 1.07 mg/day in the US, while in Korea, the mean intake was about 37 mg/day [35,36]. A similar intake of anthocyanins has been reported in Australia, where the mean intake was about 32 mg/day. Women, on average, had a higher daily intake of anthocyanins (35.4 ± 25.2 mg/d) compared with men (28.5 ± 21.8 mg/d) [37]. In Europe, the daily intake ranges from 19.8 to 64.9 mg/day for men and from 18.7 to 44.1 mg/day for women [38]. The main sources of anthocyanins in Europe are fruits such as grapes, apples, pears, berries (approximately 50%), and wines. In the United States, the intake of berries, vines, grapes, and bananas is responsible for approximately 50% of the estimated habitual intake of anthocyanins [39].

## 5. Bioavailability

The studies aiming to determine the anthocyanin blood and urine concentration levels after the ingestion of foods rich in anthocyanins suggest poor bioavailability (<1%) [40]. However, Czank et al. [41] have shown that the relative bioavailability of an isotopically labeled version of one of the most prevalent anthocyanins in food, cyanidin-3-*O*-glucoside, was 12.3% (^13^C5-labelled anthocyanin). These data and recent studies suggest that anthocyanin bioavailability may be higher than previously assumed if taking into account conjugated products, unmetabolized parent compounds, and metabolites resulting from xenobiotic and bacterial metabolism [16,40,42,43]. Moreover, the content of a meal can also influence anthocyanins’ bioavailability. There is evidence that without having other meals, the accessibility of total anthocyanins was enhanced by 10–15% when red cabbage was co-digested with the carotenoid-rich vegetables, except for carrot [44]. Therefore, a good understanding of not only their absorption, distribution, biotransformation/metabolism, and excretion in the human body but also aspects of co-digestion is essential for better understanding and interpreting their particular biological effects [22,39].

Following the digestion process, anthocyanins’ metabolism undergoes an intense variation in pH that, together with the endogenous and microbial enzymes, can lead to the reduction and hydrolysis reaction and transformation of anthocyanins into metabolites and catabolic products [16,22,39,45]. According to literature data, anthocyanins are absorbed into the bloodstream where they are transported to target tissues [23]. Evidence suggests that anthocyanins and some of their metabolites may cross the blood–brain barrier and exert actions at the molecular level, influencing signaling pathways, genetic expression, and protein function [46]. Further, the variability effect of these substances among individuals depends, first of all, on food matrix and processing, and then enzymatic levels affected by genetic factors and diet, age, and sex, and finally microbiota functionality [16,47].

## 6. Anthocyanins and Their Antioxidant and Anti-Inflammatory Activity

Anthocyanins and their metabolites, which are found in food, possess many biochemical properties, but the best-investigated effect is their antioxidants [48,49,50]. These substances can be mediated by inhibition of both the activity and production of various pro-inflammatory important substances and enzymes, such as TNF-α, nitric oxide (NO), inducible nitric oxide synthase (iNOS), cyclooxygenase-2 (COX-2), and lipoxygenase (LOX). They are also known for upregulating the production of glutamate-cysteine ligase (GCL) and of glutamate-cysteine ligase modifier subunit (GCLM), and consequently, the levels of reduced glutathione (GSH), in activated microglial cells [51,52]. Of natural substances, anthocyanins have been suggested to play an important role in the suppression of inflammation also by inhibition of nuclear factor-kappa B cells (NF-κB) activation, which, in turn, is responsible for the control of transcription of DNA, cytokine production, and cell survival [53,54]. For example, cyanidin-3-glucoside, major anthocyanin of black rice (*Oryza sativa* L.), inhibited nuclear translocation of NF-κB p50 and p65 signaling in a 5-Fluoruracil-induced oral mucositis rat model and in oral keratinocyte culture [55]. Another investigator showed that malvidin-3-glucoside, found in rabbiteye blueberry (*Vaccinium ashei*), also suppressed TNFα in human umbilical vein endothelial cells by inhibiting nuclear translocation of p65 of NF-κB [56]. Treatment with cyanidin-3-*O*-sophoroside and cyanidin-3-*O*-sambubioside from black peanut ameliorated UV-irradiated oxidative injury through the action of the nuclear factor erythroid 2-related factor 2 (Nrf2) by interaction with the NF-κB signaling pathway in human keratinocyte cells (HaCaT cells) and mice skin [57]. This finding suggested that anthocyanins from black peanut skin might regulate oxidative stress and the suppression of cell apoptosis and might be used as a potential protective agent against UV-B-induced skin damage.

These substances have the capacity to act as antioxidants, and they can mediate antioxidant effects mainly by free radical scavenging or by chelating metal ions. Mechanisms of antioxidant action include (1) their ability to reduce the release of ROS formation by inhibition of enzymes or by chelating trace elements involved in the free radical generation, (2) scavenging ROS, and (3) upregulation or protection of antioxidant defenses [58,59]. The chelation of metals seems to be crucial in the prevention of radical generation. Some of these flavonoids are capable of inhibiting the enzymes involved in ROS generation, for example, microsomal monooxygenase, glutathione S-transferase, mitochondrial succinoxidase, and dihydro-nicotinamide adenine dinucleotide (NADH) oxidase [60]. The antioxidant action of anthocyanins may also result from activation of not only antioxidant enzymes, such as catalase, glutathione peroxidase, and heme oxygenase-1 (HO-1), which have radical scavenging ability, but also by suppression of prostaglandin E2 (PGE2), which impairs T cell receptor signaling [61]. The anti-inflammatory activity of anthocyanins was evaluated by both in vitro and in vivo analysis (Table 2).

Anthocyanins may exert a pivotal role in the prevention and management of several pathologies, including cardiovascular diseases, cancer, diabetes, and degenerative diseases, which are induced by persistent oxidative stress. These substances were shown to exhibit anti-aging effects on mouse neural stem cells (mNSCs), where treatment with anthocyanins from *Ribes meyeri* improved the cellular aging phenotype, with reduced levels of ROS and aging-related P16ink4a gene expression, increased DNA synthesis phase, and lengthened telomeres. Stem cell exhaustion is an important hallmark of aging where the main reasons for aging include shortened telomeres, increased levels of ROS, activation of the senescence marker P16ink4a, and cell cycle arrest. Anthocyanin treatment may have anti-aging effects on mNSCs by promoting the formation of neurons and by increasing mNSCs proliferation and neurogenesis [62].

It is well recognized that preventing chronic inflammation may delay or prevent the development of diabetes [63]. Oxidative stress appears to play a key role in the pathogenesis of β-cell dysfunction, impaired glucose tolerance, and the development of insulin resistance in T2DM. Hyperglycemia may increase the susceptibility to lipid peroxidation in the body, which ultimately contributes to the increased incidence of atherosclerosis, a major complication of T2DM [64,65]. Therefore, the protective effects of anthocyanins against the development of both type 1 diabetes mellitus and T2DM could be mediated through anti-inflammatory actions in a variety of cell types [66,67]. The anti-diabetic effect of anthocyanins extracted from purple corn was investigated in dual-layer cell culture with Caco-2 cells, INS-1E pancreatic β-cells, and HepG2 hepatocytes cells [68]. Purple corn is rich in cyanidin, peonidin, and pelargonidin, where they reside as glycosides. Pure anthocyanin extract (100 μM) enhances insulin secretion and hepatic glucose uptake through activation of the free fatty acid receptor-1 (FFAR1) and glucokinase (GK), respectively, and potentially ameliorate T2DM comorbidities [68]. Recently, it was reported that the anthocyanin cyanidin-3-glucoside (C3G) regulated carbohydrate metabolism, inflammatory markers, and gut microbiota in a high-fat high-sucrose (HFHS)-diet-induced insulin-resistant animal model [69]. The supplementation of C3G (7.2 mg/kg/day in a diet) attenuated hyperglycemia, hypercholesterolemia, hypertriglyceridemia, and insulin resistance, while the concentrations of pro-inflammatory markers such as monocyte chemotactic protein-1 (MCP-1), and plasminogen activator inhibitor-1(PAI-1) were also reduced. Furthermore, C3G increased the *Bacteroidetes/Firmicutes* phylum bacteria ratio and the abundance of gut *Muribaculaceae* family bacteria by the reduction of the abundance of gut microbial genes involved in inflammation and enhanced gut microbial genes involved in the metabolic processes in mice receiving the HFHS diet [69].

Anthocyanins also have beneficial effects on hyperglycemia-induced endothelial dysfunction [70]. The microvascular endothelium seems to be a major target of hyperglycemic damage. Endothelial cells take up glucose passively in an insulin-independent manner and cannot downregulate the glucose transport rate when glucose concentration is elevated, resulting in intracellular hyperglycemia, which significantly affects endothelial cell biology [71]. Indeed, microvascular damage is a key early event in the development of many diabetic complications. Further, this can lead to an increase in the inflammatory response and perhaps to the expression of various pro-inflammatory cytokines and chemokines, including IL-6, IL-8, interleukin-1α (IL-1α), and TNF-α [71]. Anthocyanin-rich sour cherry extract exhibits depletion of hyperglycemia-induced ROS production in human umbilical cord vein endothelial cells, thereby alleviating oxidative stress [70]. The key molecules of the inflammatory processes caused by hyperglycemia, such as TNF-α, IL-6, IL-8, and IL-1α, were reduced. Further, the investigated extract has been reported to increase the expression of NOS and decrease the expression of various genes responsible for vasoconstriction. Hence, anthocyanins play a crucial role as immunomodulatory, anti-oxidative, and potent vasorelaxant agents.

The antioxidant activity of anthocyanins and their glycosides have been widely investigated. Findings from in vivo and in vitro research suggest that these substances may have the potential to exert an anti-inflammatory effect in vivo. Pre-clinical studies have also shown that anthocyanin supplementation has anti-cancer, anti-inflammatory, and angiogenesis activity [72,73,74,75,76,77].

## 7. Clinical Studies on Anthocyanin Interventions—An Update from the Last 5 Years

Anthocyanins have been widely substantiated by preclinical evidence as compounds with metabolic regulatory effects on LGCI that may contribute to preventing or delaying the onset of cancer, diabetes, Alzheimer’s disease, cardiovascular, pulmonary, and autoimmune diseases, but the evidence in humans is still limited. However, numerous randomized controlled trials have been conducted lately. They mostly focus on the anti-inflammatory effects of anthocyanins and their impact on the cardiovascular system and cognitive functions in both healthy and chronic disease populations (Supplementary Table S1). In this update, we present the clinical studies that provide us with the strongest evidence for the role of additional anthocyanin intakes in the manifestation of inflammation in both chronic diseases and healthy subjects.

### 7.1. Anthocyanins and Inflammation Markers Metabolic Disorders

Low-grade chronic inflammation is one of the primary mechanisms of cardiovascular diseases (CVDs) and plays an essential role in the initiation and progression of CVDs but is also related to other forms of CVDs, such as hypertension, dyslipidemia, peripheral arterial disease, coronary artery disease, and ischemic stroke. This group of disorders is still the leading cause of morbidity and mortality worldwide [86,87]. Therefore, identifying modifiable risk factors and understanding the importance of a natural food supplement that could effectively lower LGCI would contribute to the prevention and treatment of MetS, T2DM, and obesity.

The beneficial effect of anthocyanins among MetS individuals was widely investigated. In a clinical control trial, patients with MetS who consumed diets supplemented with 320 mg of anthocyanins for four weeks displayed reductions in the serum fasting blood glucose by 13%, triglyceride (TG) by 25%, and low-density lipoprotein cholesterol (LDL-C) by 33%, and attenuations in hs-CRP level by 28% among females, as compared to healthy subjects [88]. Dietary supplementation of anthocyanins (320 mg/daily) was also associated with inhibiting the expression of pro-inflammatory genes and factors related to NF-κB pathways including TNF-α (by −28% and −15%), IL-6 (−16.1% and −13.6%), IL-1A (−21.5% and −12.9%), platelet endothelial cell adhesion molecule-1 (PCAM-1) (−15% and −17.5%), and COX-2(−26% and −27%) in both MetS and control group, respectively [89]. The transcription factor NF-κB regulates cellular immune responses to infection and oxidative stress as a pivotal mediator of the inflammatory response. The activation of NF-kB leads to the induction of pro-inflammatory mediator expression, including inducible COX-2, iNOS, NO, IL-1, IL-6, TNF-α, adhesion molecules, and chemokines [90]. Anthocyanin supplementation, which was shown to have beneficial effects on inflammatory cascade, may have a role in the development of therapeutic strategies based on NF-κB inhibition.

In line with the results above, the anti-inflammatory effects of anthocyanins were also reported in pilot randomized, placebo-controlled clinical trials [91,92]. Twelve-week dietary supplementation with Açaí berry beverages decreased two biomarkers for inflammation and oxidative stress, plasma level of interferon gamma (IFN-γ), and urinary level of 8-isoprostane in MetS individuals [91]. Further, Montmorency tart cherry juice attenuated the oxidized low-density lipoprotein (OxyLDL) and soluble vascular cell adhesion molecule-1(VCAM-1) protein, which is closely associated with the progression of various immunological disorders [92,93].

Current research provides links among dyslipidemia and elevated levels of low-grade chronic inflammatory cytokines, such as interleukin IL-6, TNF-α, and CRP [94]. In a dose–response randomized control trial (RCT), 12-week anthocyanin supplementation was the most efficient and positively improved the anti-oxidative and anti-inflammatory capacity by reducing serum of IL-6 by 40%, TNF-α by 21%, malondialdehyde (MDA) by 20%, urine 8-isoPGF2α by 37%, and 8-hydroxy-2′-deoxyguanosine (8-OHdG) by 36% and significantly improving T-SOD in dyslipidemic individuals. The daily dosage of 320 mg was the most efficient [95]. It has also been well documented that obesity results in the secretion of strong pro-inflammatory and regulatory mediators, as well as cytokines or chemokines [8]. The anti-inflammatory effects of anthocyanins were also reported in patients with obesity or overweight in both short- and long-term interventions. Daily consumption of dried juçara pulp (~131.2 mg of anthocyanins) for 6 weeks reduced inflammation gene and protein expression in monocytes from obese patients [96]. Further, whole purple wheat (containing not only anthocyanins but also other phytochemicals and fiber) attenuated the inflammatory response in obese subjects, where reduction in IL-6 and TNF-α was observed [97]. Recent clinical studies that analyzed the effect of acute meal supplementation with strawberries reported divergent results concerning IL-6 levels among patients with overweight and obesity [98,99]. However, all data presented above seem to use anthocyanins as a potential tool against obesity-related sub-clinical inflammation.

In the last 5 years, there has been a limited number of clinical studies in which pre-diabetic or T2DM volunteers are supplemented with anthocyanin-rich products and different parameters in relation to diabetes and oxidative and inflammatory markers are measured [67]. Over a 4-week period, anthocyanin supplementation had beneficial effects on such pro-inflammatory molecules as IL-6, IL-18, and TNF-α in T2DM patients [100]. Consistently, these results further show that anthocyanins supplements may be useful in amelioration inflammation by favorably decreasing secretion of inflammatory biomarkers.

### 7.2. Anthocyanins and Inflammation Markers in Healthy and Physical Active Individuals

Regarding anthocyanins’ effects on lipid profile, inflammation, and oxidative stress, their effect is more noteworthy in hyperlipidemic and obese patients than in healthy subjects. This results from the fact that healthy subjects’ baseline measures of oxidative stress and inflammation are relatively low, making it more difficult to detect any changes as a result of anthocyanin supplementation. However, many clinical trials evaluated the modulation of oxidative stress biomarkers in healthy subjects, which was manifested by increasing the activities of the antioxidant enzymes and total antioxidant capacity [101,102,103,104,105].

A wide variety of anthocyanin supplements, particularly those from fruits such as berries, strawberry, açaí berries, riceberry, or other plant extracts, have been used in healthy individuals for their effects on inflammatory agents. De Liz et al. [102] determined the effect of administration of açaí juice on oxidative stress biomarkers in healthy adults. Oxidative stress was assessed, including total antioxidant capacity (TAC) and oxidative stress index (OSI), where TAC is the sum of both enzymatic and nonenzymatic antioxidants and OSI indicates the relationship between antioxidant mechanisms and oxidant concentrations [106]. Açaí juice in 4-week intervention promoted increases in TAC by 67% and attenuated OSI by 56% in healthy adults. Further, anthocyanins from açaí enhanced the erythrocytes catalase (CAT) and glutathione peroxidase (GPx) enzyme activities compared to baseline [102]. Interestingly, the concentrations of high-density lipoprotein cholesterol (HDL-c) by nearly 8% was observed, which indicated the positive effects of regular consumption of açaí juice on cardiovascular health. Similarly, one-time combination of anthocyanins and bromelain supplementation among 18 healthy adults improved TAC, oxygen utility capacity, and brachial artery flow-mediated dilation (FMD) [105]. These results suggest that anthocyanin intake may be an effective nutritional therapy for improving vascular health.

Other clinical studies were performed with anthocyanin-rich foods as sources of anthocyanins. It must be emphasized that natural sources of these substances consist of many other protective substances such as fiber or other polyphenols. Moreover, phytochemicals and vitamins found in fruits and vegetables could interact synergistically or antagonistically with anthocyanins, thereby enhancing or decreasing the antioxidant activity of these compounds [21]. In healthy individuals, intake of yogurt enriched with anthocyanins from riceberry rice (*Oryza sativa* L.) significantly increased plasma ferric reducing ability of plasma (FRAP), trolox equivalent antioxidant capacity (TEAC), and oxygen radical absorbance capacity (ORAC) and reduced plasma MDA, which is a well-known lipid peroxidation product [103].

Not all reports provide evidence of favorable effects of anthocyanin on anti-inflammatory and oxidative stress markers concentrations in healthy subjects. Some long-term randomized clinical trial reports indicated that 60 mg/day supplementation of anthocyanins for eight months among postmenopausal women produces favorable effects on glucose levels. However, no changes in inflammatory and antioxidant parameters, such as CRP, IL-6, VCAM-1, intercellular adhesion molecule-1 (IAM-1), monocyte chemoattractant protein-1 (MCP-1), or matrix metalloproteinases 2 (MMP-2) and MMP-9, were observed [107]. Similarly, no changes in plasma adiponectin, IL-6, IL-1β, MCP-1, TNFα, or CRP were observed among healthy former smokers in 12-week, 500 mg aronia extract supplementation. However, reduction in plasma total cholesterol (TC) and LDL-C was observed [108].

There has also been an interest in the properties of anthocyanins in alteration inflammation processes related to physical activity. Given these data, it is tempting to speculate that consumption of black currant nectar during eccentric exercise attenuates muscle damage and inflammation by reducing creatine kinase (markers of muscle damage) and positive changes in ORAC and IL-6 concentrations [109].

### 7.3. Anthocyanins and Neuroinflammation

It is well known that antioxidant defenses are unbalanced in mostly neurological pathologies [17,110,111]. Anthocyanins are unique neuroprotective agents that have been shown in animals to modulate neuroinflammation, and interest in their use as therapeutics for neurodegeneration has grown in recent years [111].

In a recent clinical study, anthocyanins and their metabolites appeared to ameliorate cognitive decline in aging adults, with either slight or no effects on pro-inflammatory markers. Do Rosario et al. [112] showed that 8-week anthocyanins intake decreased the serum concentrations of TNF-α in older adults diagnosed with mild cognitive impairment (MCI). However, no other inflammatory biomarkers have been found. In turn, Kent et al. [113] evaluated the protective effects of 12-week consumption of 200 mL/day of anthocyanins-rich cherry juice containing 138 mg of anthocyanins in adults over 70 years with mild-to-moderate dementia. Cherry juice improved verbal fluency, short-term memory, and long-term memory. Moreover, the intake of juice promoted a decrease in systolic blood pressure. However, inflammatory markers like CRP and IL-6 were not altered. Similarly, blueberry extract (equivalent to approximately 387 mg of anthocyanidins) intake for 12 weeks enhanced working memory and cerebral brain perfusion in healthy older adults [114]. Corresponding to Kent et al.’s [113] results, anthocyanins supplementation did not affect hsCRP. However, all authors highlighted that the short intervention length and small number of participants have been the main limitations of the studies presented above.

### 7.4. Anthocyanins and Other Inflammatory Markers Reports

Recent clinical studies have been also reported anthocyanin effects on inflammation in patients with insomnia, chronic kidney diseases, and patients after acute myocardial infarction [115,116,117]. In placebo-controlled, crossover pilot study conducted among patients with insomnia, daily consumption of cherry juice decreased the level of PGE-2 and indicated an inhibition of the enzyme indoleamine 2, 3 deoxygenase (IDO). Inhibition of IDO may decrease inflammation [118]. In addition, the sleep time was extended by 84 min among participants [117]. Interestingly, supplementation of extruded sorghum breakfast cereal combined with unfermented probiotic milk decreased the CRP and malondialdehyde serum levels and increased the TAC and SOD enzyme levels in patients with chronic kidney disease, suggesting that anthocyanin and other phytochemicals may protect from inflammation and oxidative stress in these group of patients [116]. In turn, findings from the RCT suggested that bilberries may have clinically relevant beneficial effects on patients after acute MI. However, the inflammatory markers such as hs-CRP did not change with bilberry supplementation [115].

## 8. Summary

Low-grade chronic inflammation is a key factor in the pathogenesis of many chronic diseases. Therefore, identifying modifiable risk factors that could effectively lower LGCI would contribute to the prevention of chronic disease.

This narrative review summarizes the main clinical studies in the context of the anti-inflammatory and anti-oxidative role of anthocyanins in both health and disease. Growing numbers of pre-clinical studies suggest its modulatory effect on inflammation pathways. Nevertheless, quantifying the effect of anthocyanins on inflammation in randomized control trials is hard to assess. The evidence that supports the direct anti-inflammatory role of anthocyanins is relatively scarce or of low quality. However, it supports the anthocyanins’ role in regulating inflammation. Over the past five years, at least 29 clinical studies have evaluated the relationship between anthocyanins supplementation and inflammation. However, inconsistencies between the result of RCTs and meta-analyses have been observed. The main limitations of the presented clinical data are the short length of observation, small samples, and a wide variety and dosages of anthocyanin supplements that were used. It must be underlined that anthocyanins interventions have been more widely investigated in the treatment of metabolic outcomes. Human studies have reported lower LDL-cholesterol and triglycerides and increased HDL-cholesterol. Thus, the data support an indirect and beneficial role of anthocyanins in improving LCGI.

Regarding anthocyanins’ effects on inflammatory markers, there is a need for long-term clinical trials allowing for the long preclinical phase or quantifiable progression of inflammation. Future research and intervention should also take into account the validation of the reduction of inflammatory markers by these compounds as well as other potential regulatory effects such as gut microbiota activity, which anthocyanin bioactivity depends on. Furthermore, strategies for improving techniques to evaluate gut microbiota's impact on the bioavailability of anthocyanins are needed. Additionally, a better understanding of the role of anthocyanins in inflammation could not be fully established without knowledge of the effects of treatment of pure anthocyanins. The synthesis of pure anthocyanins for research purposes remains crucial. Further translational research is necessary to understand the pharmacological actions of anthocyanins in humans.

The present review can clinicians and other health care professionals in understanding the importance of both supplemental and natural sources of anthocyanins in the LGCI and inflammatory status of patients. Anthocyanins treatment as a natural therapeutic strategy, free of side effects, could represent a useful tool in the prevention and treatment of low-grade chronic inflammation.

## Figures and Tables

**Table 1:** Content of anthocyanidins in selected foodstuffs (mg per 100 g of foodstuff); based on [28,29].

	Anthocyanidins (mg/100 g), Edible Portion						
Product	Pelar	Cyan	Delph	Peon	Petun	Malv	Total
Raspberries, black	16.69	669.01	nd	1.09	nd	nd	685.70
Plum, Illawara, (*Podocarpus elatus*)	2.47	555.72	nd	nd	nd	nd	558.19
Elderberry juice concentrate	nd	411.40	nd	nd	nd	nd	411.40
Chokeberry	0.98	344.07	0.65	0.08	2.79	1.22	349.79
Bilberries	0.00	85.26	97.59	20.45	42.69	39.22	285.21
Cowpeas, black seed cultivar, raw (*Vigna unguiculata* Subsp. *Sinensis*)	nd	94.72	94.60	11.07	27.82	34.28	262.49
Cabbage, red, raw (*Brassica oleracea* (Capitata Group))	0.02	209.83	0.10	nd	nd	nd	209.95
Service (Saskatoon) berries (*Amelanchier canadensis*)	0.00	110.58	50.38	2.96	6.27	10.59	180.78
Blueberries, cultivated (highbush), (*Vaccinium* spp.)	0.00	8.46	35.43	20.29	31.53	67.59	163.30
Black currant (*Ribes nigrum*)	1.17	62.46	89.62	0.66	3.87	nd	157.78
Blueberries, rabbiteye, (*Vaccinium* spp.)	nd	9.60	23.41	15.90	36.25	63.45	148.61
Radicchio, raw (*Cichorium intybus*)	nd	126.99	7.68	nd	nd	nd	134.67
Grapes, Concord, (*Vitis vinifera*)	nd	23.76	70.62	4.78	14.93	6.01	120.10
Sweet dessert wine	nd	nd	3.90	3.93	6.63	94.83	109.29
Blackberries (*Rubus* spp.)	0.45	99.95	0.00	0.21	0.00	0.00	100.61
Purple corn (*Zea Mays)* *	9.3	68.5	nd	20.3	nd	nd	98.1
Molucca raspberry, (*Rubus moluccanus var. austropacificus*)	4.07	90.17	nd	nd	nd	nd	94.24
Maqui (Chilean wineberry), (*Aristotelia chilensis*)	nd	22.37	66.15	nd	nd	nd	88.52
Eggplant, raw (*Solanum melongena*)	nd	nd	85.69	nd	nd	nd	85.69
Red currants	nd	65.54	9.32	0.16	nd	nd	75.02
Guajiru (coco-plum)	nd	nd	15.19	1.82	55.72	nd	72.73
Radishes, (*Raphanussativus*)	63.13	0.00	0.00	0.00	0.00	0.00	63.13
Acai berries, purple,	nd	53.64	nd	nd	nd	nd	53.64
Raspberries, (*Rubus* spp.)	0.98	45.77	1.32	0.12	0.31	0.13	48.63
Black beans, mature seeds, raw (*Phaseolus vulgaris*)	nd	nd	18.50	nd	15.41	10.61	44.52
Strawberries (*Fragaria X ananassa*)	24.85	1.68	0.31	0.05	0.11	0.01	27.01
Wheat, purple	3.41	11.07	3.20	1.81	2.34	4.02	25.85
Red table wine	nd	0.19	2.01	1.25	1.98	13.84	19.27
Pecan nuts	0.00	10.74	7.28	0.00	0.00	0.00	18.02
Pistachio nuts	0.00	7.33	0.00	0.00	0.00	0.00	7.33
Hazel nuts	0.00	6.71	0.00	0.00	0.00	0.00	6.71

* data based on [29]. Pelar—pelargonidin, Cyan—cyanidin, Delph—delphinidin, Peon—peonidin, Petun—petunidin, Malv—malvidin, nd—no data.

**Table 2:** In vivo and in vitro research update (studies published in 2020) on anthocyanins and their anti-inflammatory effects in pathological conditions.

Disorder/Substances	In Vitro or In Vivo Model		Mode of Action	References
**Adipose Tissue Inflammation**				
Delta-tocotrienol, (DT3), and tart cherry anthocyanins (TCA)	3T3-L1 adipocytes	↓	IL-6 secretion and expression from adipocytes Down-regulation of Mip2, and COX-2 mediated via the NFkB	Harlan et al. [78]
cyanidin-3-*O*-glucoside	Murine 3T3-L1 hypertrophic adipocytes		Modulating the expression of the PPAR-ɣ, Inhibiting the inflammatory pathway modulated by NF-κB	Molonia et al. [79]
**Pulmonary Artery Hypertension**				
Cyanidin-3-*O*-β-glucoside	Transforming growth factor-β1 (TGF-β1)-mediated human pulmonary arterial smooth muscle cells (SMCs), Pulmonary artery hypertension (PAH) rats	↓ ↑ ↓	IL-6, TNF-α and IL-10 SOD activity MAD Suppressive effect on PAH progression	Ouyang et al. [80]
**Diabetes**				
*Padus racemose* Anthocyanins	H2 O2 -induced rat insulinoma (INS-1) pancreatic cells damage		inhibiting the activation of p38 MAPK and NF-κB	Liu et al. [81]
**Hypercholestrolemia and Hepatic Inflammation**				
Black Raspberry (*Rubus occidentalis*)	Rats fed high-fat and high-choline diets	↓ ↓ ↓	cecal TMA and serum oxidized TMAO, TC, LDL mRNA expression of pro-inflammatory genes including NF-κB, IL-1β, IL-6, COX-2 protein expression of NF-κB and COX-2 in liver tissue	Lim et al. [82]
**Cancer**				
rice bran, cyanidin 3-glucoside	Human prostatic cancer (PC3) cells	↓ ↑	expression of Smad/Snail signaling molecules expression of cell surface protein, E-cadherin Inhibited matrix metalloproteinase-9 and NF-κB Mediating Snail/E-cadherin expression	Jongsomchai et al. [73]
*Vitis coignetiae Pulliat* (Meoru in Korea)	MCF-7 Human Breast Cancer Cells	↑	Inhibiting Akt and NF-κB activity Cisplatin (anti-cancer drug) sensitivity	Paramananthm et al. [72]
Dark Sweet Cherry (*Prunus avium)*	MDA-MB-453 breast cancer cells and athymic mice xenografted with MDA-MB-453 breast cancer cells	↑	Bax/Bcl-2 ratio Activation of MAPKs ERK1/2 and p38 Down-regulation of total oncogenic and stress-related Akt	Layosa et al. Noratto et al. [74,75]
*Vitis coignetiae Pulliat* (Meoru in Korea)	Hep3B Human Hepatocellular Carcinoma Cells		Inhibition of the activation NF-κB and suppressed the NF-κB-regulated proteins, Inhibition of proliferation, invasion, and angiogenesis	Kim et al. [76]
**Gastric Ulcer**				
Dried acai berries extract (*Euterpe oleracea*)	Ethanol-induced gastric ulcer in rats	↑ ↓	GSH content and GST and CAT activity MPO activity, TNF-a	Cury et al. [83]
**Neuroinflammation**				
*Hibiscus sabdariffa* L. (Malvaceae)	Streptozotocin-induced Alzheimer’s disease in mice	↓ ↓	TNF-α, IL-6, and IL-1β Elevated MDA and MPO Reverse up-regulation in the amyloidogenic pathway	El-Shiekh et al. [84]
Delphinidin	Alzheimer’s disease model in rats	↓ ↓	AChE, APP, and Aβ ROS overproduction in hippocampus	Heysieattalab et al. [85]
Portugal Blueberries (*Vaccinium corymbosum* L)	Mouse microglia N9 cell line	↓ ↓ ↓ ↑	Suppression of NF-kB and STAT1 NO, PGE2, COX-2 TNF-α Intracellular Production of ROS GSH	Serra et al. [51]
**Cataract**				
Cyanidin-3-*O*-glucoside	High glucose-induced lens epithelial cell (SRA01/04)		Inhibition SRA01/04 cell apoptosis Regulation of the Bcl-2/Bax ratio Suppression of NF-κB activation and subsequent Cox-2 expression	Song et al. [54]

↑—increase, ↓—decrease, Aβ—amyloid beta, AChE—acetylcholinesterase, Akt—protein kinase B, APP—amyloid precursor protein, Bax/Bcl—Bcl-2-associated X protein/B-cell lymphoma protein ratio, CAT—catalase, COX—cyclooxygenase, GSH—reduced glutathione GST—glutathione S—transferases, IL—interleukin, MAPK—mitogen-activated protein kinases, MAD—malondialdehyde, Mip2—macrophage inflammatory protein 2, MPO—metalloproteinase NFkB—nuclear-factor κB, PAH—pulmonary artery hypertension, PGE— prostaglandins, PPAR-ɣ—peroxisome proliferator-activated receptors gamma, ROS—reactive oxygen species, SOD—superoxide dismutase, STAT1—signal transducer and activator of transcription 1, TMA-trimethylamine, TMO—trimethylamine-*N*-oxide.

## Data Availability

Not applicable.

## Supplementary Materials

The following are available online. Table S1: The summary of (A) clinical trials in particular disorders and (B) meta-analyses. References [119,120,121,122,123,124] are cited in the supplementary materials.
