# Supplementary material for: Targeting Inflammation by Anthocyanins as the Novel Therapeutic Potential for Chronic Diseases: An Update
Source: Molecules. 2021 Jul 20;26(14):4380. doi: 10.3390/molecules26144380 (PMC8304181; doi:10.3390/molecules26144380)
Supplement: Supplementary file 1 [file molecules-26-04380-s001.zip › molecules-1299640-supplementary.pdf]

Supplementary Materials

Table S1. The summary of (A) clinical trials in particular disorders and (B) meta-analyses.

Study (year)	Study Design and Comparator (if applicable)	Number of participants	Drug/Substance	Dosage [mg/d]	Condition	Treatment/ use duration	Endpoints/Measures	Outcomes	Limitations
**A. Clinical trials in particular disorders**									
**INFLAMMATION AND CARDIOVASCULAR HEALTH**									
Aboonabi (2020a) [89]	Randomized control trial	47	Anthocyanin capsules Medox®, Purified anthocyanin from bilberry (*Vaccinium myrtillus*) and blackcurrant (*Ribes nigrum*)	2x 160 mg/daily	MetS and healthy subjects, aged 25-75 years	4 weeks	• Cardiometabolic risk factors<br>• Anti-atherogenicity and anti-platelet activities	• Decreased hs-CRP level (by 28%) in females<br>• Decreased serum FBG (by 13.3%)<br>• Reduction in triglyceride (by 24.9%) and LDL-C (by 33.1%)<br>• No significant differences in serum UA and HDL<br>• Decreased P-selectin by 40%	Small sample size
Aboonabi (2020b) [88]	Randomized control trial	35	Anthocyanin capsules Medox®, Purified anthocyanin from bilberry (*Vaccinium myrtillus*) and blackcurrant (*Ribes nigrum*)	2x 160 mg/daily	MetS and healthy subjects, aged 25-75 years	4 weeks	• MetS components and the expression of PPAR-γ, Nrf2, and NF-κB dependent genes	• Decreased TNF-α (-28% and -15%), IL-6 (-16.1% and -13.6%), IL-1A (-21.5% and -12.9%), PCAM-1 (-15% and -17.5%), and COX-2(-26% and -27%) in both MetS and control group respectively<br>• Decreased FBG (15.7% vs 3.2%), TG(18.2% vs -1.39%), cholesterol (33.5% vs 1.56%) and LDL (28.4% vs -15.6%), serum hs-CRP (-36.3% vs 6.25%) in the MetS compared to Control group	The moderate effect of age in the MetS group<br>Small sample size
Kim (2018) [91]	Randomized, double-blind, placebo-controlled clinical trial	37	Açaí berries (*Euterpe oleracea* Mart.)	Açaí-beverage (containing 1139 mg L-1 gallic acid equivalents of total polyphenolics 2 x325 ml/daily	Individuals with MetS	12 weeks	• Modulation of lipid and glucose-metabolism, as well as oxidative stress and inflammation	• Decreased plasma IFN-γ and urinary level of 8-isoprostane.<br>• No modification of biomarkers for lipid- and glucose-metabolism	Small sample size
Johnson (2020) [92]	Randomized, single-blind, placebo-controlled, parallel-arm pilot clinical trial	19	Montmorency tart cherries (*Prunus cerasus* L.)	2x 240 mL of tart cherry juice/daily	Patients with MetS	12 weeks	• Effects on hemodynamics, arterial stiffness, and blood biomarkers of cardiovascular and metabolic health in men and women with MetS	• OxyLDL and soluble VCAM-1 were significantly lower in Tart Cherry than Control<br>• Trend for total cholesterol to be lower in Tart Cherry.<br>• No significant changes were observed in hemodynamics, arterial stiffness, or other blood biomarkers assessed	Small sample size
Zhang (2020) [95]	Double-blind, randomized controlled trial	169	Anthocyanin capsules Medox®,	40mg (n = 44), 80 mg (n = 40), 320 mg (n = 42),	Dyslipidemic subjects aged 35–70	12 weeks	• 8-iso-PGF2α<br>• 8-OHdG<br>• MDA	• 320 mg/ day anthocyanin supplementation reduced serum IL-6 (−40%), TNF-α (−21%), MDA (−20%) and urine 8-	Blood concentrations of

	design with multi-dose interventions		Purified anthocyanin from bilberry (*Vaccinium myrtillus*) and blackcurrant (*Ribes nigrum*)	0 mg (n=43)			• T-SOD<br>• UA<br>• IL-6 IL-10<br>• TNF-α<br>• CRP	isoPGF2α (−37%) and 8-OHdG (−36%) than 80 mg/day and 40 mg/day anthocyanins, and significantly improved T-SOD.<br>• A slight reduction in serum IL-6, TNF-α, and urine 8-iso-PGF2α from the baseline was observed at 12 weeks in the group receiving 40 mg/day anthocyanins.<br>• Anthocyanins (80 mg/day) significantly reduced serum IL-6 (−20%), TNF-α (−11%) and urine 8-iso-PGF2α (−27%)	anthocyanins or their metabolites were not measured
Santamarina (2020) [96]	Randomized, double-blind, placebo-controlled trial	27	5 g of freeze-dried juçara pulp	~131,2 mg of anthocyanins	Obese adults aged 31 -59	6 weeks	• Inflammation gene and protein expression in the monocytes from obese individuals<br>• Monocyte cytokines production	• reduced TLR4, and IL-6 mRNA, the protein expression of TLR4, and MYD88 expression<br>• reduced IL-6, TNF-α, and MCP-1 production by monocytes<br>• increased IL-10 mRNA, and Ob-R protein levels<br>• reduced pIKKα/β	Short intervention Small sample size
do Rosario (2021a) [20]	Randomized, double-blind crossover clinical trial	16 (13 female, 3 male)	250 mL of Queen Garnet plum juice (source of anthocyanins-intervention group) Or apricot juice (control)	n/a	Overweight and obese older adults following a high fat high energy (HFHE) meal challenge	4 days	• BP<br>• FMD and microvascular perfusion<br>• IL-1β, IL-6 and TNF-α<br>• hs-CRP, TC and TG	• No significant correlations between baseline vascular parameters and inflammatory biomarkers<br>• significantly lower hs-CRP and IL-6 concentration 4 h postprandially<br>• no significant effects for concentration of IL-6, TNF- a and IL-1b	No measures before the start of the 4 day run-in period Small sample size Short observation period
Gamel (2020) [97]	Randomized, single-blind parallel-arm study	29	Whole purple and regular wheat	Four daily servings	Overweight and obese adults with chronic inflammation (hsCRP > 1.0 mg/l)	8 weeks	• Anthropometrics, blood markers of inflammation, oxidative stress, and lipaemia<br>• Metabolites of anthocyanins and phenolic acids	• Reduction in IL-6 and increase in adiponectin in the purple wheat (PW) group<br>• TNF-α was lowered in both groups and ferulic acid concentration increased in the regular wheat (RW) group	Not only anthocyanins but also fiber and other phytochemicals
Huang (2016) [98]	3-arm, single-blind, crossover clinical trial	14	Strawberry drink	12 g of freeze-dried strawberry powder which is equivalent to ~1 cup of fresh strawberries.	Overweight healthy adults	Strawberry drink 2 hours before a standard meal, or with the meal, or 2 hours after the meal	• Meal-induced postprandial response in inflammation and oxidative stress as well as post-meal insulin responses.	• significant reduction in postprandial glucose when strawberry was consumed before the meal compared to having the strawberry drink with the meal<br>• IL-6 was significantly lower after consuming the strawberry drink before the meal	Small sample size Single blinding
Park (2016) [99]	Randomized, single-blind, diet-controlled crossover trial	25	FDS- freeze-dried whole strawberry powder rich in anthocyanins (pelargonidin)	0g of FDS 0.04 anthocyanins mg/beverage 10g of FDS= 42.2 anthocyanins mg/beverage	Obese individuals with insulin resistance	One serving with breakfast	• The dose–response relationship of strawberries on postprandial glucose and insulin<br>• Changes in plasma anthocyanins,	• Postmeal insulin concentrations (6 h) were significantly reduced after the 40-g FDS beverage<br>• Postmeal 6 h glucose concentrations were not different	Lack of plasma data on other non-anthocyanin strawberry polyphenols and their metabolites

				20g of FDS=87.9 anthocyanins mg/beverage<br>40g of FDS= 154.5 anthocyanins mg/beverage			markers of oxidative stress, and inflammation	• Pelargonidin-glucuronide was inversely associated with mean insulin concentrations after the 20 and 40 g FDS<br>• OxyLDL was reduced after 20gFDS<br>• IL-6 was not different among treatments	No additional inflammatory marker assessment associated inflammatory
Nikbakht (2021) [100]	Open-label clinical trial	40	Anthocyanin capsules Medox®, Purified anthocyanin from bilberry (*Vaccinium myrtillus*) and blackcurrant (*Ribes nigrum*)	320 mg	Patients with type T2DM, T2DM-at-risk and healthy individuals 25–75 y	4 weeks	• DII<br>• Biomarkers of inflammation<br>• Lipid profile<br>• FBG, UA	• Reduction of IL-6, IL-18, and TNF-α in T2DM<br>• Reduction LDL in T2DM-at-risk adults but not in the T2D group<br>• Improved FBG and UA in T2DM-at-risk group	Small sample size<br>Short duration of the intervention
**INFLAMMATION AND HEALTHY ADULTS**									
Jokioja (2020) [119]	Randomized cross-over trial	17	Mashed yellow-fleshed potatoes with or without purple potato extract (PPE) rich in acylated anthocyanins and other phenolics	yellow potatoes with or without purple potato extract (PPE, extracted with water/ethanol/acetic acid) rich in acylated anthocyanins (152 mg) and other phenolics (140 mg)	Healthy men	Two potato meals and a wash-out time of at least two weeks	• Postprandial inflammation<br>• Glycemic, insulinemic and inflammatory responses	• Decrease glucose level after the meal<br>• PPE affected some of the studied 90 inflammation markers after meal; for example IL-6, IL-7, VEGF A	Other phenolics were used
Guo (2020) [101]	Randomized, double-blind, placebo-controlled trial	107	Capsules contained 17 different kinds of purified anthocyanins, (mostly delphinidin-3-O-glucosides and cyanidin-3-O-glucosides)	An increasing dosing schedule of 20, 40, 80, 160, and 320 mg	Healthy adults aged 18 to 35 y	14 days	• FPG<br>• Inflammatory biomarkers (IL-6, IL-10, and TNF-α)<br>• Oxidative markers (8-iso-PGF2a, MDA, TAOC level)	• No changes in TNF-α, MDA, TAOC levels<br>• Decreased 8-iso-PGF2a<br>• Significant decreased in FPG in the groups receiving 40, 80 160, and 320 mg/d	Healthy status of participants in this study<br>Baseline measures of oxidative stress and inflammation were relatively low
De Liz (2020) [102]	Randomized cross-over, single-blind clinical trial	30	Drink 2 X 100 mL/day of açaí or juçara juice rich in anthocyanins and unsaturated fatty acids	Açaí juice = 99.85 mg of anthocyanins / 200 ml<br>Juçara juice= 626.57 of anthocyanins / 200 ml	Healthy adults, aged between 19 and 59 y	4 weeks with a 4-week washout	• FBG, lipid profile<br>• Oxidative stress biomarkers, TAC,TOS, oxidative stress index (OSI), UA<br>• Activity of the SOD, CAT, and GPx	• Açaí and juçara juices increased the HDL-c by 7.7% and 11.4%, respectively<br>• Açaí juice intake promoted significant increases in TAC (66.7%), CAT (275.1%), GPx (15.3%), and a decrease in OSI (55.7%) compared to baseline<br>• Juçara juice intake significantly increased CAT activity (~15.0%) in relation to baseline<br>• Açaí juice increased the levels of FBG 7.3%	Absence of a control group, Uncontrolled physical activity habits throughout the study

Hillman (2021) [120]	Randomized, double-blind, placebo-controlled trial	48	1st group: Montmorency tart cherry capsules (CherryPURE®) once or twice daily<br>2nd group: The tart cherry juice once or twice daily<br>3rd group: placebo	Capsules contained 480 mg of freeze-dried tart cherries	Healthy participants	2 days	• Markers of inflammation: UA, hsCRP<br>• Oxidative capacity: ORAC	• No significant change in hsCRP or ORAC or between groups<br>• Acutely reduced levels of UA up to 8 h post ingestion (an average 8% decrease)	Used a commercially available tart cherry juice not from concentrate that also contained apple juice, which has not been analyzed for phenolic content or ORAC
Xie (2017) [108]	Randomized, placebo-controlled trial	49	500 mg aronia extract	45.1 mg anthocyanins, 35.7 mg hydroxycinnamic acids, and 41.9 mg proanthocyanidins per 500-mg dose	Healthy adult former smokers	12 weeks	• Biomarkers of cardiovascular disease risk, inflammation, and oxidative stress in former smokers<br>• Plasma lipids, blood pressure, and lipid transport genes of peripheral blood mononuclear cells	• No change in BP or biomarkers of inflammation and oxidative stress<br>• Reduction in FBG, TC by 8%, LDL-C by 11%, and LDL receptor protein in peripheral blood mononuclear cells	Small sample size<br>Former smokers with different degrees of past smoking<br>Analysis did not capture antioxidant mechanism(s) of aronia consumption
Anuyahong (2020) [103]	Randomized-crossover trial with a one-week washout period	19	Yogurt enriched with anthocyanins from Riceberry Rice (*Oryza sativa* L.)	17.4 mg of cyanidin-3-glucoside and 7.9 mg Peonidin-3-glucoside per serving	Healthy subject aged 18 to 40 y	One serving,	• Postprandial glucose level<br>• Plasma antioxidant capacity<br>• MDA	• Reduction in the postprandial glucose excursion (40.23%)<br>• Acute increase in FRAP, TEAC, and ORAC<br>• Reduction in plasma MDA	Other macro- and micronutrients may interfere
Bialasie-wicz (2018) [104]	Randomized placebo-controlled trial	63	Sour Cherries	500 g of sour cherries containing 346.5 mg of total anthocyanins or 500 g of anthocyanin-free apples everyday	Healthy subject	30 days	• Inflammatory response and oxidative stress: LBCL(reflecting oxidants generation by circulating phagocytes)	• Sour cherries inhibited median resting LBCL (by 29.5% and 33.7%) and resting and agonist (fMLP)-induced LBCL (by 24.7% and 32.3%) after 30-day consumption and after 10-day wash-out	Short duration<br>Analysis only fasting blood samples
Estévez-Santiago (2019) [107]	Randomized control trial	72	Anthocyanins, xanthophylls, or both groups of bioactives together	Group A anthocyanins (60 mg/d);<br>Group X xanthophylls (6 mg lutein + 2 mg zeaxanthin/d)<br>Group A+X anthocyanins (60 mg/d) + xanthophylls (6 mg	Postmenopausal Women	8 months	• Inflammatory, antioxidant, and cardiometabolic parameters	• No changed in SBP, DBP, BP, CRP, IL-6, VCAM-1, IAM-1, MCP-1-1 or MMP-2, and MMP-9<br>• Decresed plasma glucose levels and the plasma metabolomic profile by all three dietary supplementations<br>• No synergistic effect between the two groups of bioactives	Lack of placebo group<br>No dietary recommendations or control

				lutein + 2 mg zeaxanthin/d)					
Pekas (2021) [105]	Randomized, double-blind, placebo-controlled clinical trial	18	Anthocyanins from hawthorn berry extract(*Crataegus* spp.)and tart cherry powder (*Prunus cerasus*), and bromelain	Two capsules with a total of 465, 480 and 400 mg of hawthorn extract, tart cherry powder and bromelain, respectively	Healthy adults<br>Mean age 24	One serving,	• Impacts on endothelial function, BP, TAC, and OUC<br>• Brachial artery FMD, and fatigability were measured	• Increased FMD, TAC<br>• Reduced SBP<br>• Improved OUC<br>• Tissue saturation and oxygenated Hb significantly increased, while deoxygenated Hb significantly decreased	Mixes of substances have been used
**NEUROINFLAMMATION**									
Kent (2017) [113]	Randomized controlled trial	49	Anthocyanin-rich cherry juice	200 ml/day of cherry juice (intervention arm) (138 mg of anthocyanins) or 200 ml/day of commercially prepared apple juice (control arm)	Older adults (+70 year) with mild-to moderate dementia	12 weeks	• Cognitive function<br>• BP<br>• Anti-inflammatory effects	• CRP and IL-6 not altered<br>• Improvements in verbal fluency, short-term memory and long-term memory<br>• Reduction in systolic SB	Small sample size<br>Short intervention length
do Rosario (2021b) [112]	Randomized, double-blind, controlled clinical trial	31	The high-anthocyanins juice containing queen garnet plum	250 mL fruit juice daily<br>(1) 201 mg<br>(2) 47 mg<br>(3) 0 mg	Older participants with MCI mean age 75.3	8 weeks	• microvascular function,<br>• 24-h ABP<br>• inflammatory biomarkers	• Decreased TNF- α in group that received the higher dose of anthocyanins<br>• Anthocyanins did not alter other inflammatory biomarkers, microvascular function or blood pressure parameters	The average mean intake of anthocyanins in the low dose group in the usual diet of participants was similar to the intervention
Bowtell (2017) [114]	Randomized, double-blind, controlled clinical trial	26	30 mL blueberry concentrate	387 mg anthocyanidins (34 mg malvidin, 108 mg cyanidin, 41 mg pelargonidin, 63 mg peonidin, 86 mg delphinidin, and 55 mg petunidin)	Healthy older	12 weeks	• Brain perfusion, task-related activation, and cognitive function<br>• 5 cognitive domains: attention, memory, verbal fluency, language, and visuospatial abilitie<br>• Brain perfusion was determined using an arterial spin labelling technique, and blood biomarkers of inflammation and oxidative stress	• Improvement in working memory<br>• Increased resting-state cerebral perfusion in the parietal and occipital lobes<br>• Serum of BDNF concentration was not affected<br>• Supplementation did not affect hsCRP	Small sample size<br>Blood samples were not taken in a fasted state which may introduce significant variation and confounding, since feeding status has been shown to affect serum BDNF concentration
**INSOMNIA**									

Losso (2018) [117]	Placebo-controlled, balanced, crossover pilot study	8	Cherry juice	240 ml twice a day	Subjects >50 years of age and insomnia	2 weeks	• Sleep was evaluated by polysomnography and 5 validated questionnaires<br>• Serum IDO, the kynurenine to tryptophan ratio, and PGE-2<br>• In vitro, Caco-2 cells were stimulated with IFN-γ and the stimulates inflammation was measured.	• Increased sleep time by 84 minutes<br>• Sleep efficiency increased on the Pittsburgh Sleep Quality Index<br>• The serum kynurenine to tryptophan ratio decreased, as did the level of PGE-2<br>• In vitro, cherry juice procyanidin B-2 dose-dependently inhibited IDO	Small sample
**INFLAMMATION AND PHYSICAL ACTIVITY**									
Arevström (2019) [115]	Randomized, open-label, clinical trial	50	Freeze-dried bilberry (*Vaccinium myrtillus*)	40 g/d, equivalent to 480 g fresh bilberries	Patients after acute myocardial infarction	8 weeks	• Cardiovascular risk markers and exercise capacity	• No difference in hsCRP<br>• The mean 6-minute walk test distance increased significantly more in the bilberry Ex vivo OxyLDL was significantly lowered in the bilberry<br>• TC an LDL no differ	open-label design is methodologically weaker
Hutchison (2016) [109]	Randomized, placebo, control trial	16	Black currant nectar (BCN)	16 oz of BCN or a placebo twice a day	College students	8 days	• Symptoms of exercise-induced muscle damage<br>• Blood markers of muscle damage, inflammation and ORAC	• Consumption of BCN reduced CK levels<br>• The change in IL-6 was higher in the placebo group post exercise<br>• The change in ORAC levels was higher in the treatment group at 48 h post exercise	Small sample size
McCornick (2016) [121]	Randomized, double-blind, controlled clinical trial	9 male	Tart Montmorency cherries (*Prunus Cerasus*)	90 mL daily of tart Montmorency CJ concentrate	Well-trained male Water Polo players	6 days	• Markers of inflammation and oxidative stress (IL-6, CRP C, UA, F2-IsoP)	• Lack of difference observed in the blood markers between groups	The anthocyanin concentration of both the commercial and placebo supplement used was not confirmed
**CHRONIC KIDNEY DISEASES**									
Lopes 2018) [116]	Randomized, simple blind, clinical study	58	Extruded sorghum breakfast meal	n/a	Patients with chronic kidney disease	7 weeks	• Inflammation and oxidative stress markers	• decreased CRP, MDA<br>• increased the TAC and SOD enzyme	Mixes of substances (tannin, anthocyanin, and dietary fiber and probiotic)

B. META-ANALYSES

Study (year)	Study Design and Comparator (if applicable)	Number of trials (subjects)	Drug/Substance	Dosage [mg/d]	Condition	Treatment/ use duration	Endpoints/Measures	Outcomes	Limitations
Sangsefidi (2018) [122]	Meta-analysis of randomized controlled trials	7 trials (489 subjects)	purified anthocyanins or anthocyanin-rich extract supplementationPurified anthocyanins, elderberry, chokeberry, *Arctostaphylos* L. and aronia berry extracts	Dose of anthocyanins from 45.1 to 640 mg/d	Healthy and unhealthy participants (Patients with hyperlipidemia, Pre-hypertensive men)	3 to 12 weeks	• CRP levels	• Anthocyanins had no significant impact on CRP levels regarding healthy participants, patients and types of anthocyanins	Various assay methods used to determine CRP
Rahmani (2019) [123]	Meta-analysis of randomized controlled trials	7 trials (286 subjects)	Mean dose of aronia supplementation of 228 ml (juice) or mg (supplement) per day (100–500 ml or mg/day)	n/a	MetS, handball players, healthy individuals, patient after Myocardial Infraction	Mean duration of the interventions was 16 weeks (4 to 24 weeks)	• changes in lipid profile, SBP and DBP, • inflammation biomarkers: CRP, IL-6, and TNF-α	• No significant effect on SBP, CRP, IL-1 and TNF-α • Increaseed high-density HDL and DBP. • Reducted TGs levels when dose of Aronia was increased to 300 mg/day • Reduction in total cholesterol and LDL	Results did not show any difference between consumption level of aronia juice and aronia supplementation
Fallah (2020) [124]	Meta-analysis of randomized controlled trials	32 trials (1744 subjects)	Anthocyanins supplements, and anthocyanins derived from fruit	Dose of anthocyanins from 32 to 1323 mg/d	MetS, obese, prediabete, diabetes, and healthy individuals	Duration of the interventions 3 to 24 weeks	• Markers of systemic and vascular inflammation	• significantly decreased levels of CRP, IL-6 ,TNF-α, IAM-1 and VCAM-1 • Increased level of adiponectin • No change in levels(IL-1β and P-selectin • Higher doses of anthocyanins (>300 mg/day) significantly decreased levels of CRP, IL-6, TNF-α, and VCAM-1	Different sources of dietary anthocyanins that vary in composition as well as the contents of other bioactive compounds

8-iso-PGF2α— 8-isoprostaglandin F2α, 8-OHdG— 8-hydroxy-2′-deoxyguanosine, ABP— ambulatory blood pressure, BDNF— brain-derived neurotrophic factor, BP— blood pressure, CAT— catalase, COX-2—cyclooxygenase-2, DBP— diastolic blood pressure, DII— dietary inflammatory index F2-IsoP— F2-Isoprostane, FBG— fasting blood glucose, FMD— flow-mediated dilation, fMLP-LBCL— fMLP-induced luminol-enhanced whole blood chemiluminescence, GPx— glutathione peroxidase, Hb— hemoglobin, HDL— high-density lipoprotein, hs-CRP— high sensitivity C-reactive protein, IAM-1— intercellular adhesion molecule-1, IFN-γ— interferon gamma, IL—interleukin, LBLC— luminol-enhanced whole blood chemiluminescence, LDL-C— low-density lipoprotein cholesterol, MCI— mild cognitive impairment, MCP-1— monocyte chemotactic protein 1, MetS— metabolic syndrome, MDA— malonaldehyde, MMP— matrix metalloproteinase, MYD88— myeloid differentiation 88, n/a—not available or not applicable, NF-κB— nuclear-factor κB, Nrf2— nuclear factor erythroid 2-related factor 2, Ob-R— leptin receptors, ORAC— oxygen radical absorbance capacity, OSI— oxidative stress index , OUC— oxygen utility capacity, OxyLDL— oxidized low-density lipoprotein, PCAM-1— platelet endothelial cell adhesion molecule-1, PGE-2— prostaglandin E2, pIKK— phosphorylated inhibitor of κB kinase, PPAR-γ— peroxisome proliferator-activated receptors gamma, PPE— purple potato extract, SBP— systolic blood pressure, SOD— superoxide dismutase, T2DM— type 2 diabetes mellitus, TAC— total antioxidant capacity, TEAC— Trolox equivalent antioxidant capacity, TOS— total oxidant status , TG— triglyceride, TLR4— toll-like receptor 4, TNF-α— tumor necrosis factor-α, T-SOD— total superoxide dismutase, UA— uric acid, VCAM-1— vascular cell adhesion molecule-1, VEGF— vascular endothelial growth factor
